# Pain alleviation and QOL improvement of MR-guided focused ultrasound surgery (MRgFUS) treatment for painful medial compartment of knee osteoarthritis

**DOI:** 10.1186/2050-5736-3-S1-P7

**Published:** 2015-06-30

**Authors:** Motohiro Kawasaki, Syota Oda, Hiroshi Kondo, Masashi Izumi, Tomonari Kato, Masahiko Ikeuchi, Takahiro Ushida

**Affiliations:** 1 Kochi Medical School, Nankoku, Japan; 2 Aichi Medical University, Nagakute, Japan

## Background/introduction

A major symptom of knee osteoarthritis (OA) in the elderly is chronic knee pain, which has a significant effect on patients’ quality of life (QOL). Although total knee arthroplasty (TKA) is the validated and reliable treatment for alleviating refractory knee pain, this is often not an option for patients with poor health status or unwillingness to undergo major surgery. Therefore, alternative approaches to alleviate knee pain other than conventional treatments are necessary. Now, we are performing a prospective, non-randomized, single-arm study to evaluate the safety and efficacy of MRgFUS using the ExAblate 2100 conformal bone system (InSightec Ltd.) in the treatment of pain resulting from medial compartment of knee osteoarthritis.

## Methods

The study protocol was approved by the institutional review board of Kochi Medical School. Eleven patients (3 males and 8 females with a mean age of 78 years) who had medial knee pain and tenderness resistant to other conservative treatments for more than 6 months were included. All patients had Kellgren-Lawrence grade-III or IV varus knee OA. We performed a single-session treatment for more painful unilateral knee joint in each patient. Prior to MRgFUS, patient underwent local anesthesia with 0.75% ropivacaine to the treated sites. Sonications from a transducer fixed to the medial side of the knee were applied just around the osteophyte of medial femorotibial joint. They are then monitored at 1 week, 1, 3, 6 months and 1 year for treatment-related complications, levels of the worst knee pain in the past 24 hours using numerical rating scale (NRS) pain score, performance-based functional outcomes using Timed Up and Go (TUG) test and health-related QOL measures using the Western Ontario and McMaster Universities Arthritis Index (WOMAC) and EuroQol 5D (EQ-5D) questionnaires. MRI and CT were also evaluated after the treatment.

## Results and conclusions

Responders, defined as a 50% or greater decrease in NRS score compared with pre-treatment, were 8 patients one month post-treatment. In three non-responders, one patient underwent TKA. NRS pain score of the treated knee significantly decreased from 6.4 +/- 1.4 (mean +/- SD) before the treatment to 3.2 +/- 1.5 (p<0.002) at the final follow-up visit (3.5mos: 1 - 12mos) in 10 patients other than one treated with TKA. Time to perform the TUG test was significantly shorter at the final visit than pre- treatment. We also observed a significant improvement in the WOMAC scores (from 42.8 to 19.0: p<0.02) and EQ-5D scores (from 0.580 to 0.744: p<0.004). There were no obvious complications associated with the treatment. However, changes in MRI intensity signals on the treated sites were observed in all patients. In conclusion, MRgFUS treatment has the possibility to provide an improvement in patients’ quality of life, as well as the noninvasive pain management of osteoarthritic knees.

